# CRISPR/Cas9-mediated loss of *FERMT3* reveals kindlin-3–independent neutrophil activity in LAD-III

**DOI:** 10.70962/jhi.20260086

**Published:** 2026-10-21

**Authors:** Steven D.S. Webbers, Felipe Rojas-Rodriguez, Angela A.F. Gankema, Imran Khan, Judy Geissler, Anton T.J. Tool, Rupert Öllinger, Robin van Bruggen, Teunis J.P. van Dam, Suzanna C. Fagerholm, Markus Moser, Taco W. Kuijpers

**Affiliations:** 1 Department of Research, Sanquin Blood Supply Foundation, Amsterdam, Netherlands; 2 Landsteiner Laboratory, Amsterdam UMC, University of Amsterdam, Amsterdam, Netherlands; 3 Department of Molecular and Integrative Biosciences, Faculty of Biological and Environmental Sciences, University of Helsinki, Helsinki, Finland; 4 Institute of Molecular Oncology and Functional Genomics, School of Medicine, Technical University of Munich, Munich, Germany; 5 Institute of Experimental Hematology School of Medicine and Health, Technical University of Munich, Munich, Germany; 6 Center for Translational Cancer Research (TranslaTUM), School of Medicine and Health, Technical University of Munich, Munich, Germany; 7 Department of Pediatric Immunology, Rheumatology & Infectious Diseases, Emma Children’s Hospital, Amsterdam UMC, University of Amsterdam, Amsterdam, Netherlands

## Abstract

Mutations in human FERMT3 (encoding kindlin-3 protein) impair β_2_ integrin activity in neutrophils from patients with leukocyte adhesion deficiency III (LAD-III). Yet kindlin-3’s contribution to neutrophil integrin affinity regulation and function remains unclear. Using CD34^+^ human hematopoietic stem and progenitor cells (HSPCs) differentiated into neutrophils, we investigated how CRISPR/Cas9-mediated kindlin-3 deletion affects neutrophil characteristics. CRISPR/Cas9 targeting *FERMT3* resulted in an ∼80-bp deletion within exon 2, impairing protein production. *FERMT3* KO neutrophils retained normal in vitro development and phenotype with similar surface marker expression, nuclear morphology, and expansion potential compared with untreated cultured neutrophils. Loss of kindlin-3 abolished zymosan-induced NADPH oxidase activity, recapitulating the ROS production defect of LAD-III neutrophils. However, although basal adhesion was reduced, stimulus-induced integrin activity remained largely intact. *FERMT3* KO neutrophils retained fMLP-driven chemotaxis while showing impaired migration in response to C5a. Together, these findings show that disruption of kindlin-3 largely recapitulates the LAD-III phenotype while exerting some effector functions upon stimulation without intact kindlin-3.

## Teaser

Mutations in FERMT3 cause LAD-III by impairing integrin function in neutrophils. Using CRISPR/Cas9, we generated FERMT3 KO cultured neutrophils with normal in vitro granulopoiesis, transcriptomic profiles, and recapitulated defective zymosan-induced ROS production. Basal adhesion and chemotaxis were reduced, whereas stimulus-induced integrin activity remained largely intact, suggesting kindlin-3–independent neutrophil activity.

## Graphical Abstract

**Figure:**
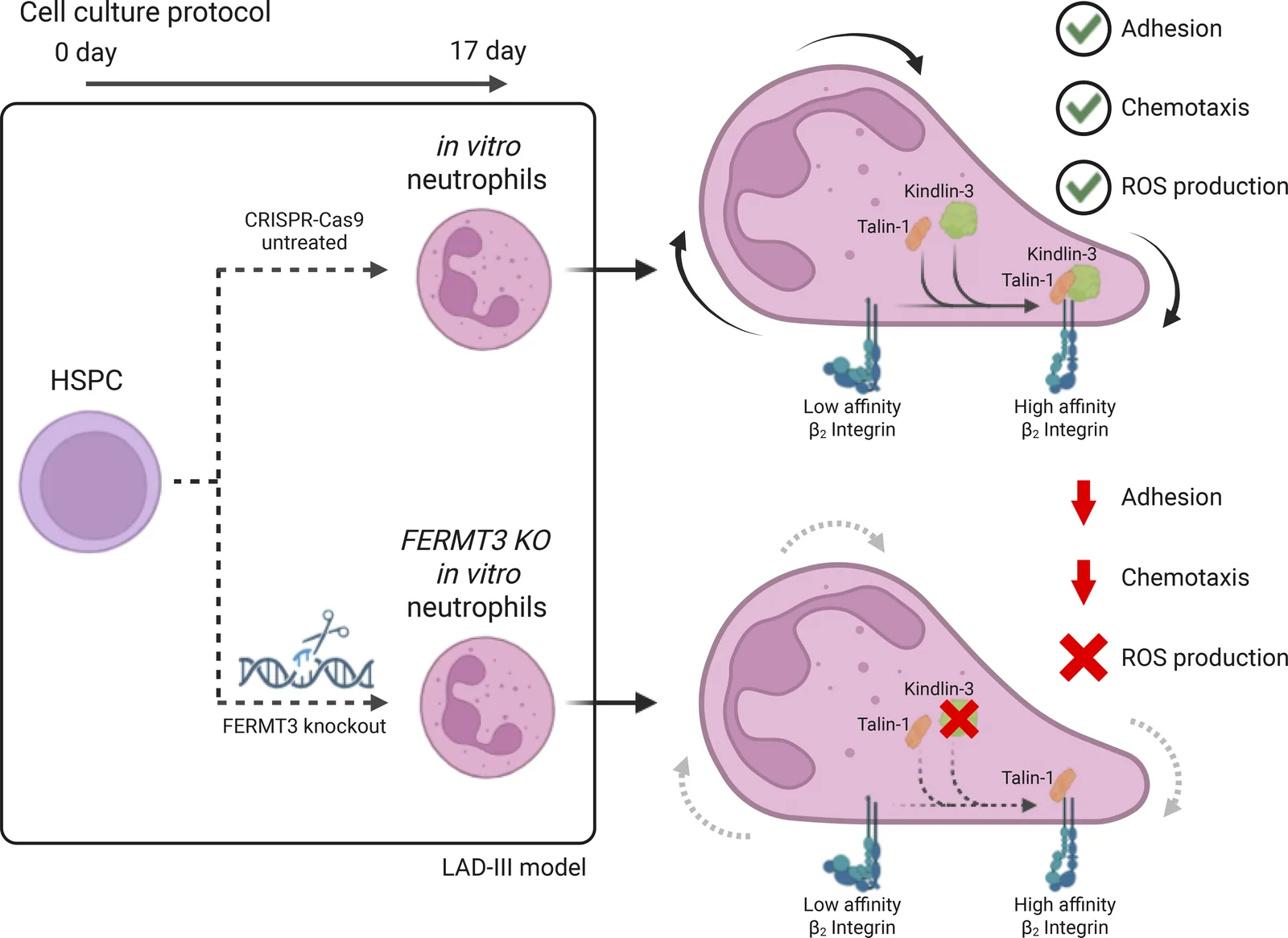

## Introduction

Neutrophils are innate immune cells acting against bacterial and fungal pathogens, modulating immune responses and contributing to the resolution of inflammation (1). Neutrophil deficiency or dysfunction can cause severe, life-threatening illness in early childhood, typically manifesting as bacterial and fungal infections. This is exemplified by LAD-I and leukocyte adhesion deficiency III (LAD-III) patients, in which defective neutrophil adhesion impairs trans-endothelial migration and migration toward the site of infection (2, 3, 4, 5, 6, 7, 8). As a consequence, neutrophils tend to remain in circulation, resulting in neutrophilia, recurrent severe infections, and increased risk of sepsis in early infancy (2, 4).

Genetically, LAD-III follows an autosomal recessive pattern. Until now, 31 different mutations within the FERMT3 gene have been described in ∼51 families (4, 5, 9, 10, 11, 12). Most are nonsense or frameshift mutations that result in strongly reduced gene transcription or instable truncated kindlin-3 proteins. For instance, a subset of mutations map to the Pleckstrin homology domain, which mediates membrane localization and phosphoinositide binding (13). Although missense variants are less common, recent work has described a *FERMT3* mutation (c.412G>A, p.E138K) that destabilizes the compact FERM domain, resulting in poor kindlin-3 expression (14).

Kindlin-3 is primarily expressed in the hematopoietic lineage and is required for integrin heterodimer activation in human neutrophils (15). This activation is mediated by the coordinated recruitment of kindlin-3 and talin-1, which facilitate the transition of β_2_-integrins from a low- to high-affinity conformation (16, 17, 18, 19, 20). The high-affinity conformation facilitates linking integrins to actin, thereby enabling neutrophil adhesion, mobility, and chemotaxis (18, 19, 20). This interaction depends on the non-catalytic F0 domain of kindlin-3, which is involved in kindlin-3–driven integrin activation and localization (21, 22, 23, 24). In LAD-III, neutrophil integrin expression is typically preserved, but loss of kindlin-3 disrupts the final step of β_2_-integrin activation, resulting in defective adhesion and extravasation from the bloodstream (9, 25). Dissecting how loss of kindlin-3 affects neutrophil function and investigating the dependence of integrin activation on kindlin-3 is challenging, due to the limited availability of primary patient material and inability to manipulate mature neutrophils in vitro (26). Murine models recapitulate the main characteristics of LAD-III and have been instrumental in defining kindlin-3 function in leukocytes (11, 27). However, key aspects of neutrophil biology differ between species; for example, murine neutrophil recruitment depends largely on CD11a/CD18, whereas in humans it is driven by CD11b/CD18 (17). This limits the extent to which mechanistic conclusions from mice can be extrapolated to LAD-III patients.

To address this limitation, we previously established a neutrophil culture system by differentiating CD34^+^ human hematopoietic stem and progenitor cells (HSPCs) toward neutrophils, yielding cells that closely resemble polymorphonuclear neutrophils (28). Here, we make use of this culture system to generate kindlin-3–deficient neutrophils by CRISPR-Cas9–mediated KO of *FERMT3*. We designed guides targeting exon 2, encoding the F0 domain of kindlin-3, expecting this lesion would impair kindlin-3 protein expression and limit β_2_-integrin activation. Our primary goal was to determine whether targeting exon 2 of FERMT3 in cultured neutrophils recapitulates the cellular phenotype observed in LAD-III patients.

## Results

### Generation of *FERMT3* KO human cultured neutrophils

**Figure 1.**
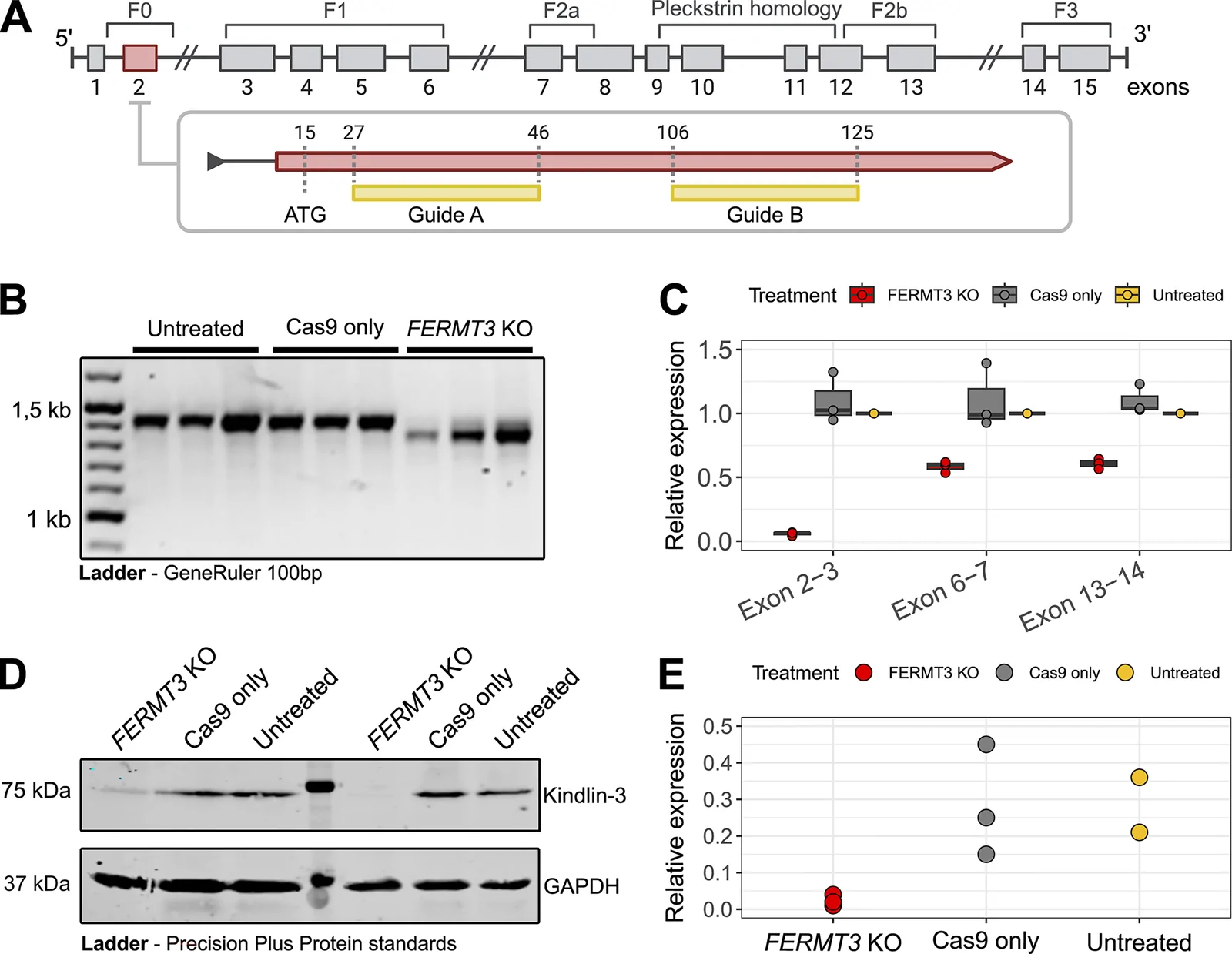
**Characterization of FERMT3 KO human CD16** ^**+**^ **cultured neutrophils. (A)** Schematics of the structure of *FERMT3*, including the location of the most representative protein domains and the location of the two gRNAs used for CRISPR-Cas9. The schematics of exon 2 include the ATG codon at position 15 of exon 2. **(B)** Electrophoresis gel showing the exons 1–7 RT-PCR amplicon of *FERMT3* KO, Cas9-only, and untreated cultured neutrophils. **(C)** qPCR quantification of the relative expression of exons 2–3, 6–7, and 13–14 for CD16^+^ fractions of *FERMT3* KO and Cas9-only cultured neutrophils. Relative expression was estimated compared with the abundance of GUS in each sample separately (*n* = 3). **(D)** Representative SDS-PAGE western blot gel for kindlin-3 and GAPDH in CD16^+^ fractions of *FERMT3* KO, Cas9-only, and untreated cultured neutrophils. **(E)** Quantification of the western blot intensity for kindlin-3 relative to the intensity of GAPDH in cultured neutrophils. Values correspond to the quantification of the SDS-PAGE available in Fig. S1; FERMT3 KO and Cas9-only *n* = 3, and untreated *n* = 2. Source data are available for this figure: SourceData F1.

**Figure S1.**
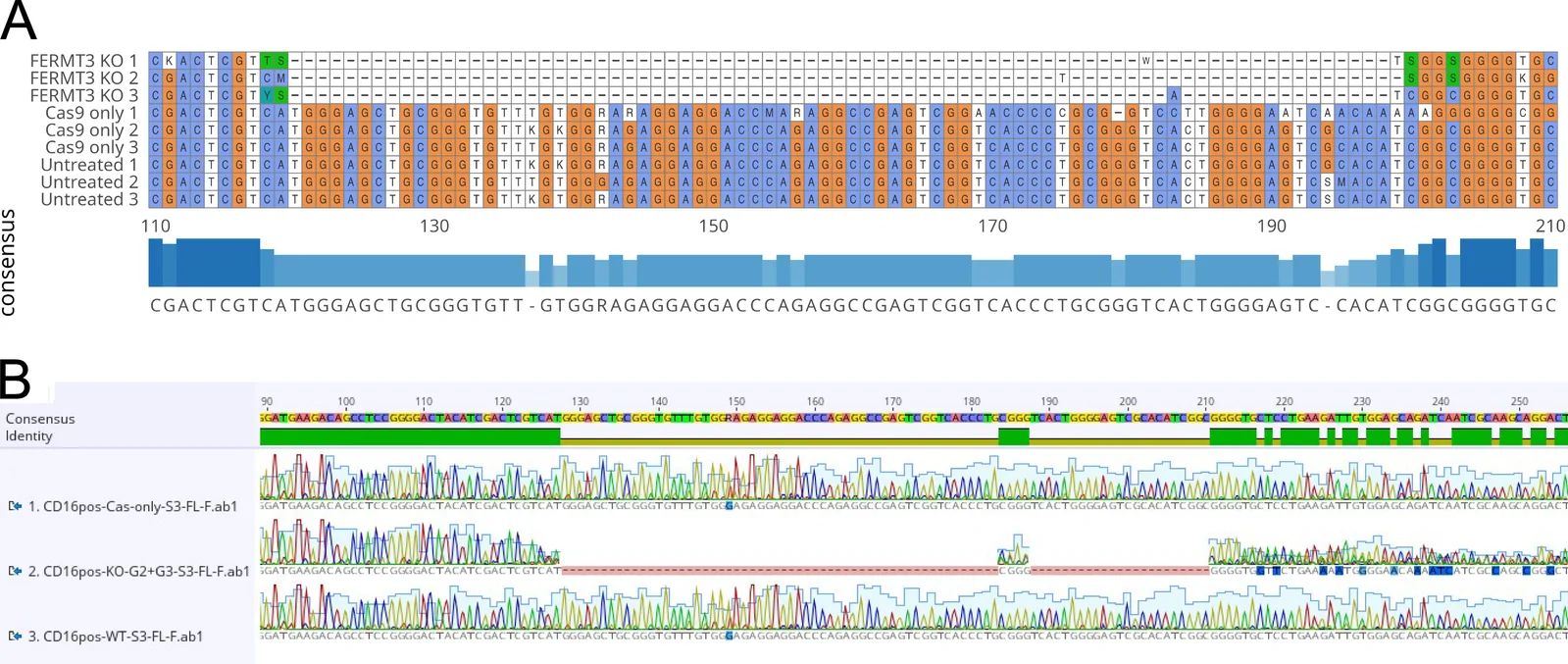
**Characterization of *FERMT3* KO human CD16** ^**+**^ **cultured neutrophils. (A)** Multiple alignment of Sanger sequencing of DNA obtained from CD16^+^ fractions of *FERMT3* KO, Cas9-only, and untreated cultured neutrophils treated with two gRNAs. Sequencing was performed on independent samples per treatment (*n* = 3). **(B)** Representative chromatogram showing the deletion occurring in exon 2.

CD34^+^ HSPCs were isolated from granulocyte colony-stimulating factor (G-CSF)–mobilized peripheral blood of healthy donors and differentiated into neutrophils over 17 days of culture (28). We generated a human cell model of LAD-III by knocking out *FERMT3* in CD34^+^ HSPCs, which were differentiated into neutrophils. We first sought to determine whether the CRISPR-Cas9 treatment resulted in the loss of *FERMT3* using two Cas9 guides targeting exon 2 at positions indicated in Fig. 1 A. Both guides, described in Table S1, were delivered by electroporation to HSPCs, with Cas9-only electroporated cells serving as the editing control. For the characterization of the most mature neutrophils in our cultures, we selected CD16^+^ fractions by magnetic-activated cell sorting (MACS). Chromatograms of cDNA were used as qualitative evidence of the deletion, with relative single-sequence traces in untreated and Cas9-only control samples (Fig. S1). Compared with cDNA sequences of untreated and Cas9-only treated cultured neutrophils, three fractions of CD16^+^*FERMT3* KO cultured neutrophils obtained from independently edited bulk culture showed ∼80-bp deletion within exon 2 (Fig. S1). We confirmed this using PCR, which showed a reduction of ∼100 base pairs within exons 1–7 in *FERMT3* KO cultured neutrophils DNA (Fig. 1 B). To elucidate the effects of exon 2 targeting on *FERMT3* mRNA, we quantified the expression of exon 2 and two additional *FERMT3* regions downstream of the CRISPR-Cas9 target site (Fig. 1 C). Transcript levels were normalized and expressed relative to β-glucuronidase (GUS), with three independent samples per condition. Quantitative PCR (qPCR) revealed efficient deletion of the targeted fragment within exon 2 in *FERMT3* KO relative to untreated cultured neutrophils (median [interquartile range; IQR]: 0.06 [0.05–0.07]) and compared with Cas9-only treatment (median [IQR]: 1.02 [0.98–1.17]). Relative expression of exon pairs 6–7 and 13–14 was diminished by 50% in *FERMT3* KO (median [IQR]: 0.6 [0.57–0.61] and 0.61 [0.59–0.63], respectively) compared with Cas9-only treatment. To assess the effect of deleting exon 2 at the protein level, we performed kindlin-3 immunoblotting in CD16^+^ fractions (Fig. 1, D and E). The monoclonal antibody used targets the epitope located at amino acid positions 156–170 of the canonical protein sequence, and the abundance of kindlin-3 was expressed relative to GAPDH, where only the samples in the same SDS-PAGE gel were considered. This revealed that simultaneous treatment with both guide RNAs (gRNAs) resulted in abrogation of kindlin-3 in CD16^+^ cultured neutrophils after 17 days of culture, although mixed populations likely remained after differentiation (∼10% residual exon 2).

### Deletion of exon 2 in *FERMT3* does not affect granulopoiesis in vitro

**Figure 2.**
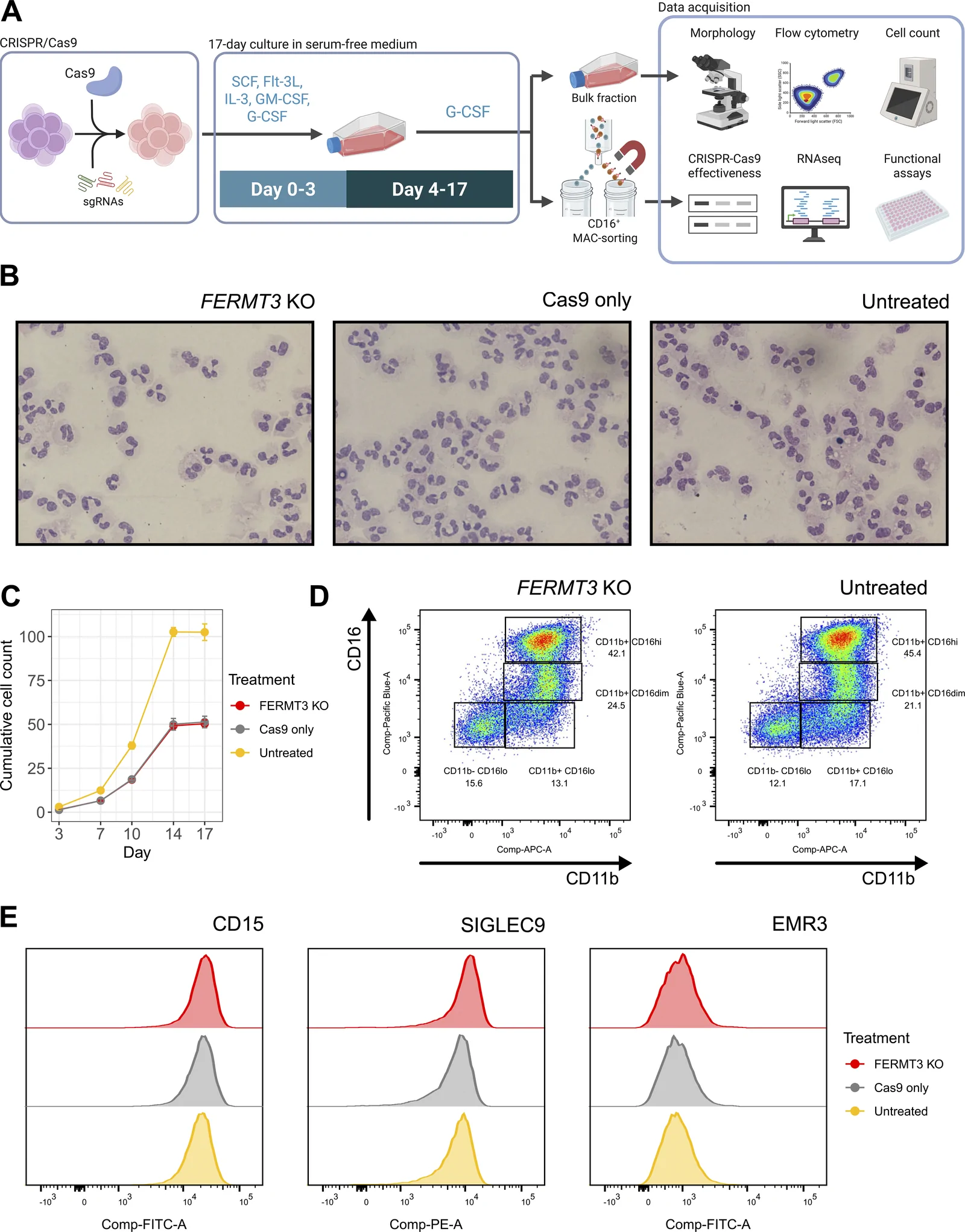
**FERMT3 KO cultured neutrophils retain normal in vitro development and neutrophil phenotype. (A)** Schematic overview of the experimental pipeline. HSPCs were subjected to CRISPR-Cas9 editing, followed by a 17-day differentiation culture to generate neutrophils in vitro. Mature cells were subsequently isolated by MACS and processed for data acquisition. CRISPR-Cas9 effectiveness, RNA-seq, and functional analyses were performed in CD16^+^ culture neutrophil fractions, while cellular morphology, cumulative cell counts, and surface marker expression were measured in the bulk fraction. These measurements were performed after 17 days of culture, unless specified otherwise. **(B)** Cytospins of bulk fractions of *FERMT3* KO, Cas9-only, and untreated cultured neutrophils. Cells were stained with May–Grünwald Giemsa staining. **(C)** Cumulative expansion of bulk fractions of *FERMT3* KO, Cas9-only, and untreated cultured neutrophils throughout the 17 days of culture (*n* = 6). Measurements were obtained after 3, 7, 10, 14, and 17 days of culture as previously described (28). **(D)** Flow cytometry expression of CD11b and CD16 surface markers of bulk *FERTM3* KO and untreated cultured neutrophils. **(E)** Flow cytometry of CD15, SIGLEC-9, and EMR3 surface marker expression on bulk fractions of *FERMT3* KO (grey), Cas9-only (pink), and untreated (yellow) cultured neutrophils.

**Figure S2.**
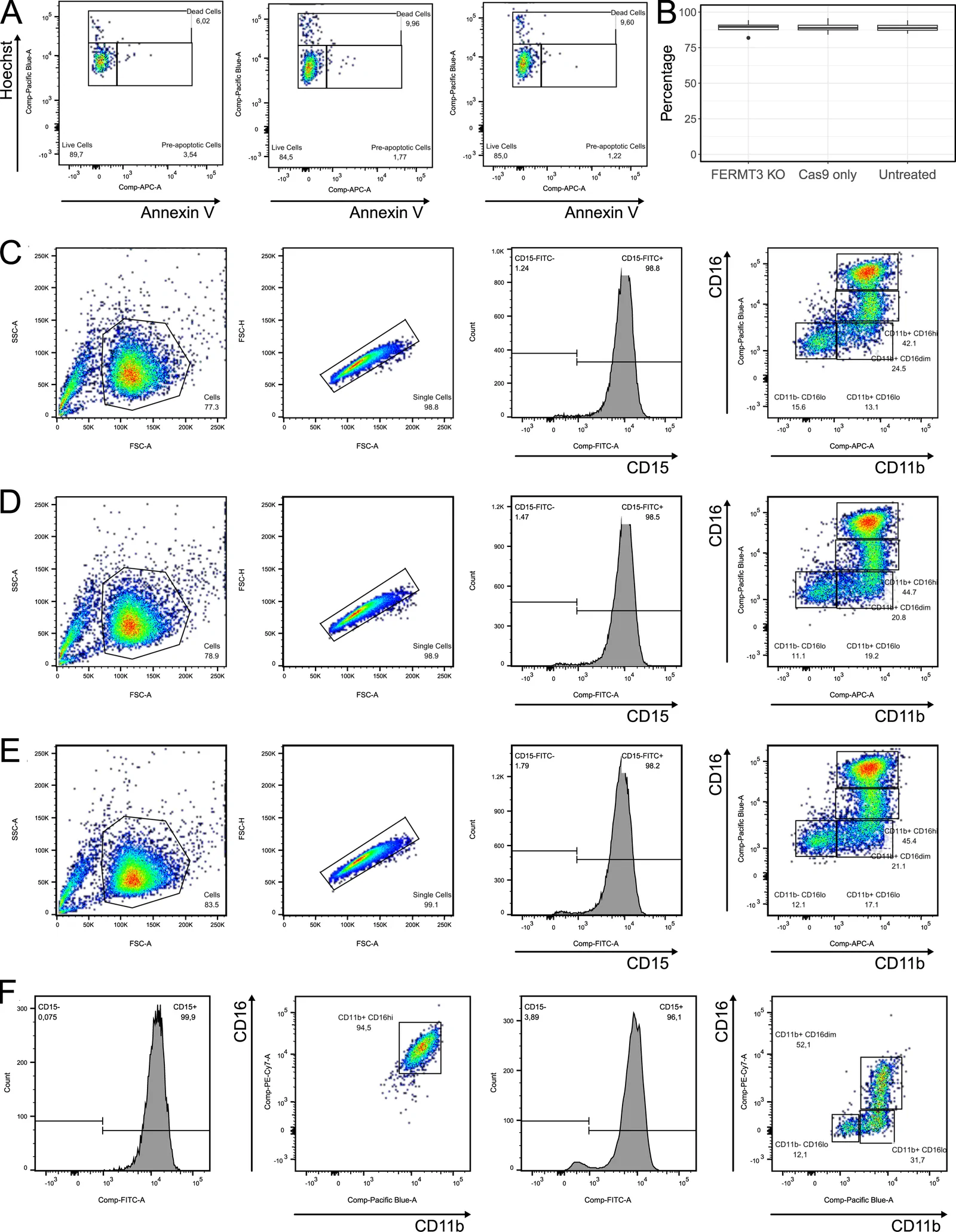
**Gating strategies used for the characterization of bulk fractions of FERMT3, Cas9-only, and untreated cultured neutrophils. (A)** Representative Annexin V FACS for bulk cultured neutrophil fractions on day 17 using Annexin V–APC-conjugated and Hoechst. **(B)** Box plot of the percentage of living cells observed for FERMT3 KO, Cas9-only, and untreated cultured neutrophils based on Annexin V. *n* = 4 for each condition. **(C)** Gating strategy for Kindlin-3 KO cultured neutrophils on day 17 of culture. **(D)** Gating strategy for Cas9-only transfected cultured neutrophils on day 17 of culture. **(E)** Gating strategy for untreated cultured neutrophils on day 17 of culture. **(F)** Representative plots from flow cytometric analysis after CD16^+^ MACS sorting of cultured neutrophils for CD16^+^ fraction (left) and CD16-depleted fraction (right).

We determined whether kindlin-3 loss impaired in vitro granulopoiesis, which was determined by nuclear morphology, cumulative expansion, and expression of maturation of surface markers by flow cytometry (Fig. 2 A). The typical neutrophil polymorphonuclear morphology after 17 days of culture remained largely unchanged between bulk *FERMT3* KO cultured neutrophils and untreated or Cas9-treated cultures (Fig. 2 B). To assess whether the CRISPR/Cas9-mediated gene editing of *FERMT3* impaired differentiation in vitro, we quantified the cumulative expansion (Fig. 2 C). Cumulative cell count was calculated by normalizing the number of cells at each time point from day 0 to day 17 of differentiation to the number of cells at day 0. At the end stage of differentiation, the *FERMT3* KO cultured neutrophils’ cumulative expansion (*n* = 6, mean ± SD: 50.4 ± 2.2) closely resembled the Cas9-only treated (*n* = 6, mean ± SD: 51.3 ± 3.3). While these conditions showed 50% lower cumulative expansion compared with untreated cultured neutrophils, which is likely due to electroporation stress, no differences in programmed cell death based on Annexin V were observed between conditions at day 17 (Fig. S2). Additionally, surface marker expression of CD11b and CD16 showed no major differences between KO cultured neutrophils and the untreated and Cas9-only treated counterparts in bulk fractions (Fig. 2 D and Fig. S2). Moreover, similar results were obtained when measuring CD15, SIGLEC-9, and EMR3 neutrophil maturation markers (Fig. 2 E). Surface marker expression was quantified by flow cytometry on day 17 using the antibody panel detailed in Table S2.

**Figure S3.**
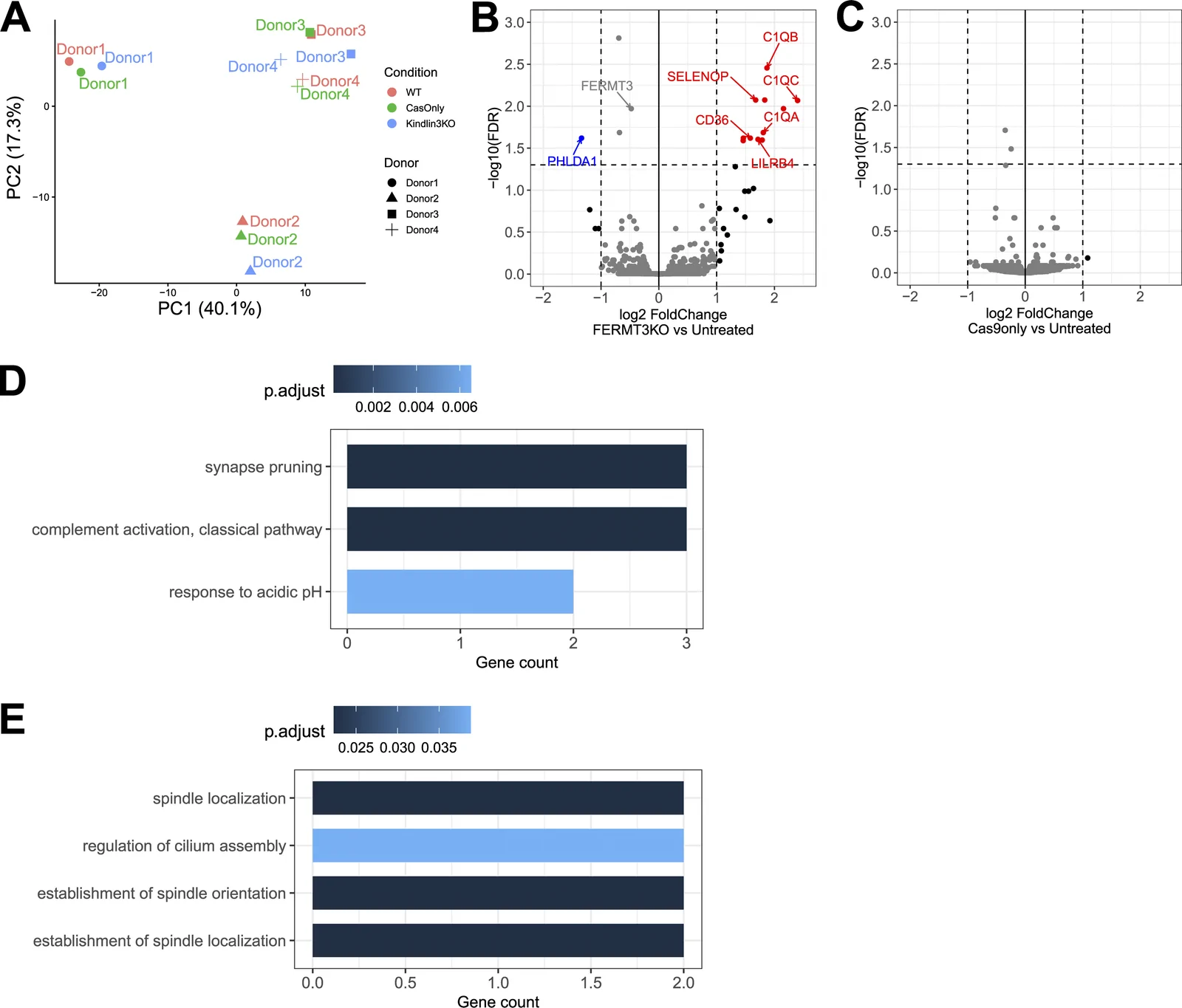
**Effect of depleting kindlin-3 on the transcriptomic profile of cultured neutrophils. (A)** Principal component analysis of FERMT3 KO (blue), Cas9-only (green), and untreated (red) cultured neutrophils obtained from four different HSPC donors. **(B)** Volcano plot of differential gene expression in FERMT3 KO cultured neutrophils compared with untreated cultured neutrophils (*n* = 4 per condition). **(C)** Volcano plot of differential gene expression in Cas9-only cultured neutrophils compared with untreated cultured neutrophils (*n* = 4 per condition). **(B and C)** Genes were considered statistically significant after Benjamini–Hochberg multiple testing correction, adjusted P < 0.05. Genes were separated according to effect size between those with |log_2_ fold-change|>1. The selected group of genes is labeled (SELENOP, CD36, LILRB4, C1QA, C1QB, C1QC, CDKN1A, FERMT3, CCL2, and PHLDA1). **(D)** Bar plot of Gene Ontology (GO) terms found in the overrepresentation analysis associated with the 12 genes found differentially expressed in FERMT3 KO cultured neutrophils when compared with untreated cultured neutrophils. **(E)** Bar plot of GO terms found in the overrepresentation analysis associated with the 32 off-target genes identified for both gRNAs used for CRISPR treatment.

We aimed to test whether loss of kindlin-3 affects the expression of genes that are not directly related to neutrophil differentiation or function. We compared the transcriptomes of in vitro–differentiated *FERMT3* KO and untreated cultured neutrophils. Bulk RNA sequencing (RNA-seq) was performed on MACS-sorted CD16^+^ fractions from four independent samples per condition on day 17. Principal component analysis revealed that HSPC donor identity was the dominant source of variation rather than the CRISPR/Cas9 treatment (Fig. S3). Differential expression was done using edgeR with Benjamini–Hochberg correction for multiple testing (29). Only 12 genes were found to be differentially expressed (Log2-fold change > |1| and false discovery rate [FDR] <0.05) when comparing mature CD16^+^ fractions of *FERMT3* KO with the same fraction of untreated cultured neutrophils (Fig. S3; Table S3). Among these, we observed upregulation of genes with a predominant monocytic signature (*GPNMB*, *CD36*, *C1QA*, *C1QB*, and *C1QC*).

To investigate any off-target effects, we used CRISPOR (30) and identified 32 predicted off-target sites for the two gRNAs. Of these, 4 were exonic and 28 intronic, and none were associated with integrin activity (Fig. S3) or were differentially expressed between *FERMT3* KO and untreated cultured neutrophils (Table S4).

### Loss of *FERMT3* selectively impairs human neutrophil motility and oxidase activity

**Figure 3.**
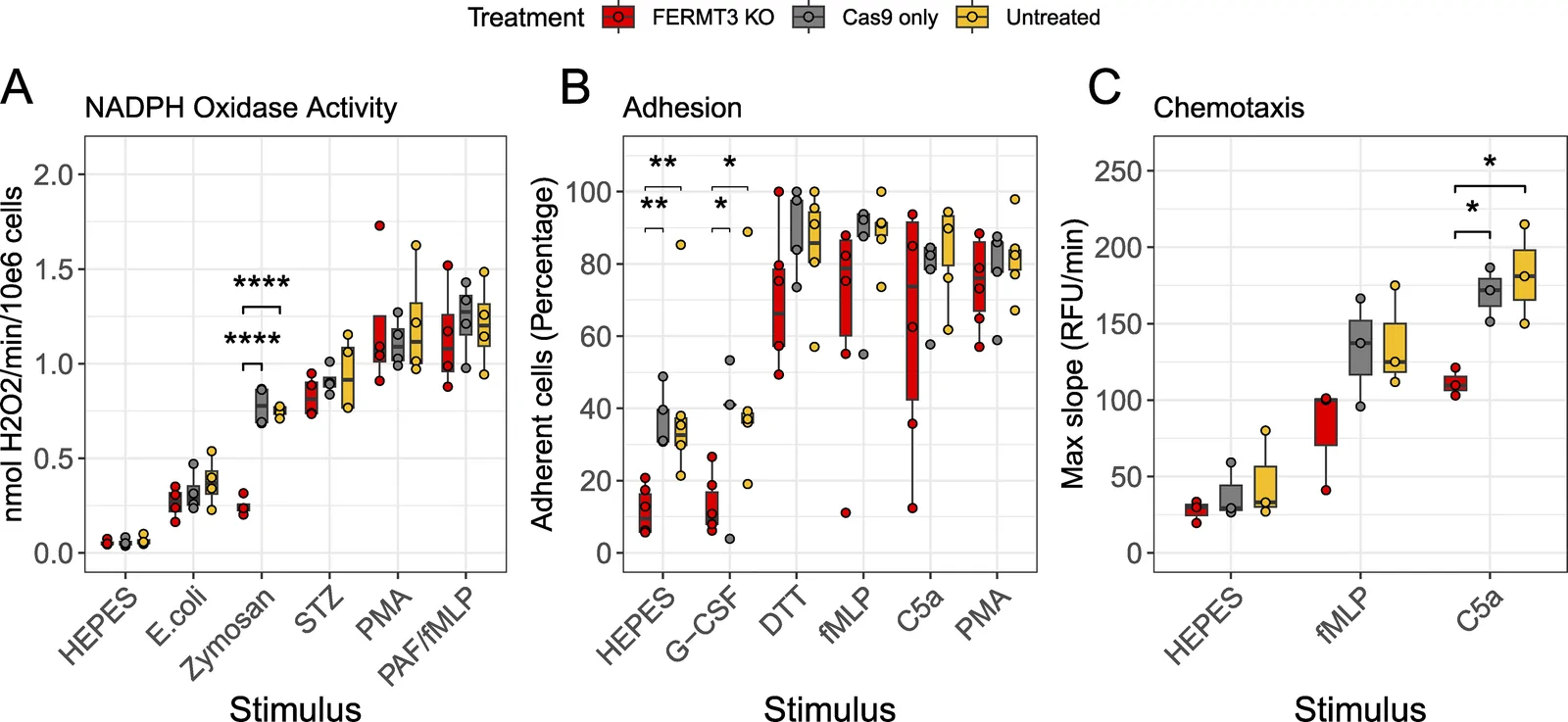
**Functional characterization of FERMT3 KO CD16** ^**+**^ **cultured neutrophils.** All assays include *FERMT3* KO (red), Cas9-only (grey), and untreated (yellow) cultured neutrophils. **(A)** NADPH oxidase activity was measured as extracellular peroxide production in untreated cells (HEPES) and after stimulation with opsonized *E. coli*, zymosan, STZ, PMA, and PAF/fMLP. Overall comparisons independent per treatment were computed using an ANOVA test, and pairwise comparisons were computed using Tukey HSD (data likely to be normally distributed; Shapiro–Wilk test adjusted P > 0.05 for all groups). *n* = 4 samples per treatment group. **(B)** CD11b/CD18-mediated adhesion assay showing the percentage of adherent cells per condition. Overall comparisons independent per treatment were computed using Kruskal–Wallis, and pairwise comparisons were computed using the Dunn test (data likely not normally distributed; Shapiro–Wilk test adjusted P ≤ 0.05 in at least one group). For Cas9-only, we included *n* = 5 samples, while *FERMT3* KO and untreated cultured neutrophils included *n* = 6 samples. **(C)** Chemotactic potential of cultured neutrophils based on movement through filters with a pore size of 3 μm. Overall comparisons independent per treatment were computed using an ANOVA, and pairwise comparisons were computed using Tukey HSD (data likely to be normally distributed; Shapiro–Wilk test adjusted P > 0.05 for all groups). We included *n* = 3 samples per treatment group. All comparisons were considered statistically significant at a nominal P value of 0.05 and labeled as: *P < 0.05, **P < 0.01, and ****P < 0.0001.

To assess whether targeting the exon encoding the F0 domain of kindlin-3 recapitulates the LAD-III patient phenotype, we evaluated key neutrophil effector functions that are typically affected in such patients (i.e., ROS production, CD11b/CD18-mediated adhesion, and neutrophil motility). Phagocytosis was not explicitly tested because the NADPH oxidase burst response to unopsonized zymosan is dependent on CD11b/CD18 (i.e., CR3), which relies on integrin activation and concomitant uptake (3, 31). Functional assays were performed on MACS-sorted CD16^+^ fractions on day 17 of differentiation using three to four independent samples per condition. CD16^+^*FERMT3* KO cultured neutrophils showed decreased NADPH oxidase activity, measured by Amplex Red, when stimulated with unopsonized zymosan compared with CD16^+^ Cas9-only and untreated cultured neutrophils (Fig. 3 A; Tukey honestly significant difference [HSD], P < 0.05). No differences in NADPH oxidase activity were observed upon stimulation with opsonized *Escherichia coli*, serum-treated zymosan, phorbol 12-myristate 13-acetate (PMA), and platelet-activating factor and N-formyl-methionyl-leucyl-phenylalanine (PAF/fMLP) (one-way ANOVA, P > 0.05). Under basal conditions (HEPES) and after G-CSF stimulation, adhesion to polystyrene plates was largely abolished in CD16^+^*FERMT3* KO cultured neutrophils compared with CD16^+^ Cas9-only and CD16^+^ untreated cultured neutrophils (Fig. 3 B; Dunn, P < 0.05). Addition of dithiothreitol (DTT) and stimulation with fMLP and C5a resulted in similar adhesion of *FERMT3* KO relative to CD16^+^ untreated cultured neutrophils (Kruskal–Wallis, P > 0.05), suggesting kindlin-3 is not indispensable for neutrophil adhesion, as we had seen before in neutrophils of LAD-III patients (26). Nonetheless, in the case of fMLP and C5a, the proportion of adherent cells exhibited considerable variability across experiments and donors. Chemotaxis assay, measured as the number of migrating cells per minute using a FluoroBlok Transwell insert, revealed that, when stimulated with C5a, CD16^+^*FERMT3* KO cultured neutrophils showed lower migration capacity (Fig. 3 C; Tukey HSD, P < 0.05). In contrast, only a minor decrease in chemotaxis was observed when stimulated with fMLP (ANOVA, P > 0.05).

## Discussion

In this study, we generated kindlin-3 KO neutrophils derived from CD34^+^ HSPCs to establish an in vitro system that mimics the cellular phenotype observed in LAD-III. This was accomplished by CRISPR/Cas9 gene editing using two gRNAs targeting exon 2 of the *FERMT3* gene, which resulted in a shortened and less abundant transcript that did not produce detectable kindlin-3 protein. Knocking out *FERMT3* seemingly did not affect neutrophil expansion potential, cellular morphology, or transcriptomic profile after 17 days in culture. We further show that these cultured neutrophils approximate the CD11b/CD18-dependent phenotype found in LAD-III patients.

In vitro granulopoiesis based on surface marker expression is not affected by the absence of kindlin-3, which is in line with normal granulopoiesis observed in LAD-III patients. Similarly, we showed that these KO cultured neutrophils display minor transcriptomic changes after 17 days of culture. Some of the differentially expressed genes exhibited predominantly monocyte-associated expression, considered rather a characteristic of our in vitro system, as shown before (28). The low number of differentially expressed genes and exonic off-target effects confirms that the functional differences observed are likely not confounded by underlying gene expression variation but rather a consequence of kindlin-3 loss. Furthermore, these *FERMT3* KO cultured neutrophils showed significantly lower NADPH oxidase activity upon stimulation with unopsonized zymosan, which is one of the diagnostic tests for LAD-III patients (3, 26). Collectively, these data suggest that CRISPR-Cas9 targeting exon 2 of *FERMT3* in HSPCs is a viable alternative to modeling LAD-III in vitro, resulting in cultured neutrophils with seemingly intact granulopoiesis.

In vitro cultured *FERMT3* KO neutrophils showed reduced, but not absent, static adhesion and retained measurable chemotactic capacity. This suggests that these neutrophils display kindlin-3–independent adhesion and chemotaxis activity upon stimulation. This is in agreement with previous observations of kindlin-3–independent activity in LAD-III patient neutrophils (26). Although baseline static adhesion was observed in control and G-CSF–stimulated CD16^+^ cultured neutrophils, likely reflecting prolonged exposure to G-CSF over the 17-day differentiation period, this baseline adhesion was slightly reduced in the absence of kindlin-3. This absence of adhesion is consistent with impaired low-affinity integrin binding observed in LAD-III patients (3). In contrast, the limited decrease in activation-linked static adhesion upon addition of DTT or stimulation with PMA corroborates that integrin activation can occur independent of kindlin-3. Likewise, the chemotactic response to C5a and, to a lesser extent, fMLP was reduced but not abolished in kindlin-3–depleted neutrophils. This aligns with previous evidence showing that priming of LAD-III neutrophils can partially restore static adhesion to polystyrene, reaching levels similar to healthy controls after treatment with fMLP (26). Together, our data further support that neutrophils can exert functional migratory properties despite loss of kindlin-3. However, caution is warranted because the variability in adhesion following C5a stimulation and in chemotaxis after fMLP stimulation may reflect donor heterogeneity as observed in the transcriptomic profile.

After CRISPR-Cas9 targeting exon 2 of *FERMT3*, we still detected residual *FERMT3* mRNA expression of exons downstream of the cut site, showing that our CRISPR-Cas9 editing resulted in alternative transcripts. This was further supported by the small differences in *FERMT3* transcript abundance observed in the RNA-seq data, suggesting that shorter *FERMT3* isoforms, lacking the regions encoding F0 and F1 domains, may arise from predicted cryptic start sites downstream of our target site (i.e., exon 5). We cannot exclude that these predicted isoforms are translated to a limited extent. However, the F0 domain that is critical for integrin activation will be missing, consistent with our model recapitulating the kindlin-3–independent LAD-III cellular phenotype and function. This is in agreement with evidence showing that genomic variants affecting early exons, encoding the F0 domain, are sufficient to cause LAD-III (5).

Our system of generating cultured neutrophils by deleting exon 2 of *FERMT3* does not come without limitations. While our study does not include exhaustive characterization of in vitro neutrophil function, we prioritized assays that are routinely used in clinical practice of LAD-III patients. Furthermore, although the disruption of β2-integrin and kindlin-3 interaction in murine models has been reported to induce strong transcriptional changes in dendritic cells and improved neutrophil survival (32), we did not detect comparable transcriptomic changes in our in vitro–generated neutrophils. This discrepancy may reflect differences between species but could also be attributed to prolonged G-CSF exposure during differentiation. Because we sample cells only after 17 days of culture, we cannot determine whether *FERMT3* depletion induces transcriptional changes during in vitro granulopoiesis. Future studies should combine longitudinal sampling with deeper transcriptomic and proteomic profiling to define the mechanisms underlying kindlin-3–independent neutrophil functionality.

Taken together, we provided evidence that *FERMT3* exon 2 deletion resulted in cultured neutrophils functionally mimicking LAD-III neutrophil phenotype without affecting granulopoiesis in vitro. Furthermore, these results support the notion that LAD-III patients can indeed exert neutrophil functionality independent of kindlin-3. We envision that this system might serve to test therapeutic alternatives for LAD-III patients and further improve our understanding of integrin biology in human neutrophils.

## Materials and methods

### Study approval

All donors provided written informed consent. The sample collection for donors was approved by the Sanquin internal Ethical Advisory Board (Privacy Declaration 1909-0176) and according to the Declaration of Helsinki. HSPCs were obtained from anonymized donors, and sex was not considered as a biological variable. HSPCs mobilized into the peripheral blood were obtained from residual leukapheresis material from healthy donors at the Amsterdam UMC, Academic Medical Center, Amsterdam, Netherlands.

### Cell culture and neutrophil isolation

HSPCs were isolated from mobilized peripheral blood by MACS with CD34-antibody beads (Miltenyi Biotec), cryopreserved, and were recovered for 1 day after thawing from cryopreservation. Day 0 to day 3 media consisted of supplemented Stemline-II (Sigma-Aldrich) medium. This included IL-3 (10 ng/ml, STEMCELL Technologies), G-CSF (30 ng/ml, Neupogen, clinical grade), granulocyte-macrophage colony-stimulating factor (GM-CSF, 10 ng/ml, PeproTech EC), Flt-3L (250 ng/ml, Stem Cell Technologies), and stem cell factor (SCF, produced in-house). From day 3, full media changes with Stemline-II supplemented with G-CSF (30 ng/ml, Neupogen, clinical grade) were performed every 3–4 days until 17 days of culture. Morphology and surface marker expression were measured in the complete bulk fraction. For all other functional analyses and efficiency of CRISPR-Cas9 treatment, CD34^+^ HSPC-derived neutrophils were sorted by MACS with CD16-antibody beads (Miltenyi-Biotec) according to the manufacturer’s protocol.

### gRNA and gene editing

gRNA selection and gene editing were performed as previously described (33). Briefly, synthetic CRISPR RNA (crRNA) sequences were selected based on high Doench and Wang scores and ordered from Integrated DNA Technologies together with trans-activating CRISPR RNA (tracrRNA) (Table S1). On the first day of CD34^+^ HSPC culture, cells were transfected with ribonucleoprotein (RNP) complexes consisting of guides and Cas9 protein, or Cas9 protein only. Guides targeting *FERMT3* were made by annealing 2.25 μl crRNA sequence 2 and 2.25 μl crRNA sequence 3 with 2.25 μl tracrRNA in equimolar concentrations of 0.2 nmol/ml for 5 min at 95°C in a PCR machine. crRNA/tracrRNA constructs were kept at room temperature, covered in aluminium foil for 20 min. Subsequently, 10 min of incubation with Cas9 (15 μg/μl, in-house production with *E. coli*) was performed. Cells were resuspended in electroporation buffer (Lonza, formerly known as Amaxa), mixed with the RNP complex solution, and subjected to electronic shock using the Lonza 4D-nucleofector (Lonza Bioscience). Cultured neutrophils treated only with Cas9 were subjected to electronic shock, while untreated cells were not. Cells were recovered in Stemline-II media, centrifuged at 300 × *g* for 5 min, and cultured until day 3 in fresh day 0 to day 3 media, which consisted of supplemented Stemline-II (Sigma-Aldrich) as described above. The number of cells was quantified using a TTC cell counter and analyzer (Roche Innovatis) on 20 μl of cell suspension. Culture continued for 17 days, as previously described (28). Off-target prediction was performed for both gRNAs using CRISPOR (30) against the GRCh38 human genome and dbSNP148 build with the default PAM matrix, excluding intergenic hits.

### qPCR

CD34^+^ HSPC-derived *FERMT3* KO neutrophils and those only treated with Cas9 were washed thrice with ice-cold PBS, resuspended in 600 μl of RNA Plus buffer (Qiagen RNeasy plus kit, 74134; Qiagen) supplemented with 1% β-mercaptoethanol, and stored at −80°C. DNA-free RNA was isolated with the Qiagen RNeasy Plus kit (74134; Qiagen) according to the manufacturer’s protocol. Primers were designed based on the *FERMT3* canonical sequence (NM_031471.6) and purchased from Integrated DNA Technologies (Coralville). cDNA was synthesized with the Superscript III first-strand synthesis system for RT-PCR (18080051; Invitrogen). qPCR reaction was done with a Luna Universal qPCR Master Mix (M3003; New England Biolabs), and amplification was performed on a 7500-I real-time PCR system (sds7500fast, cat. no.: 4406984; Applied Biosystems). qPCR data were normalized to the expression of *GUS* signal.

PCR amplification product was confirmed with Sanger sequencing using the BigDye Terminator (v1.1) in the 3730XL genetic analyzer (Applied Biosystems) and processed in the ABI Prism software (Applied Biosystems). No deconvolution was performed because of low-quality superimposed sequences downstream of the protospacer adjacent motif of guide 1. Multiple sequence alignment was performed using Geneious Prime 2023 (v.2.1; Biomatters).

### Flow cytometry

Flow cytometry data were measured using the BD FACS Canto II flow cytometer (BD Biosciences) and analyzed using FlowJo software (Version 10.7, Tree Star). In total, nine different antibodies were used in flow cytometry (Table S2). Cells were washed twice with ice-cold PBS + 0.5% human serum albumin (HSA) at 4°C by centrifugation at 300 × *g* for 5 min in v-bottom 96-well plates. Pellets were resuspended in antibody mixes and incubated for 15–20 min at 4°C, covered with aluminium foil. Afterward, cells were washed thrice with ice-cold PBS + 0.5% HSA, resuspended in ice-cold PBS + 0.5% HSA, and covered with aluminium foil at 4°C until measured. Apoptosis of cultured neutrophils was measured with Annexin V–APC conjugated (BD Biosciences) together with Hoechst (Thermo Fisher Scientific).

### May–Grünwald Giemsa staining

50,000 cells were spun at 1,000 RPM for 10 min (Shandon Cytospin II Cytocentrifuge) onto 76*26-mm glass microscope slides. The slides were air-dried and subsequently stained for 5 min in May–Grünwald (SKU 1014245000; Merck), 15 min in phosphate buffer, and 30 min of Giemsa (SKU 1092042500; Merck) solution. Slides were rinsed between steps with deionized water and air-dried.

### Immunoblot analysis

Cell lysates were prepared so that 20 μl contained 1.0*10^6^ cells. Samples were separated on 7.5% or 10% SDS-PAGE gels made in-house and subsequently transferred to nitrocellulose membranes by wet-transfer (Schleicher & Schuell). Membranes were blocked with 5% nonfat milk in tris-buffered saline with tween 20 (TBS-T, 0.1%) (Elk Campina). Kindlin-3 antibody was obtained from Dr. Markus Moser (Technical University of Munich, Munich, Germany, in-house production, rabbit, 1:500), and GAPDH antibody from Invitrogen (1:1000, mouse). Secondary antibodies were donkey anti-mouse-IgG IRDye 800CW and donkey anti–rabbit-IgG IRDye 680RD (LI-COR Biosciences). Quantification of bound antibodies was performed on an Odyssey Infrared Imaging system (LI-COR Biosciences).

### RNA-seq and data analysis

Library preparation for bulk sequencing of poly(A)-RNA was done as described previously (34). Four samples for each of the conditions (FERMT3 KO, Cas9-only, and untreated cultured neutrophils) were included. Barcoded cDNA of each sample was generated with a Maxima RT polymerase (Thermo Fisher Scientific) using an oligo-dT primer containing barcodes, unique molecular identifiers (UMIs), and an adaptor. 5′-Ends of the cDNAs were extended by a template switch oligo (TSO), and full-length cDNA was amplified with primers binding to the TSO site and the adaptor. NEB UltraII FS kit was used to fragment cDNA. After end repair and A-tailing, a TruSeq adapter was ligated, and 3′-end-fragments were finally amplified using primers with Illumina P5 and P7 overhangs. The library was sequenced on a NextSeq 500 (Illumina) with 65 cycles for the cDNA in read1 and 19 cycles for the barcodes and UMIs in read2. Data were processed using the published Drop-seq pipeline (v1.0) to generate sample- and gene-wise UMI tables (35). The reference genome (GRCh38) was used for alignment. Transcript and gene definitions were used according to GENCODE v38. The edgeR package was used for normalization, principal component analysis, and differential expression analysis (29). A fully paired design where donor was included as a blocking factor was used in the differential expression model (∼ donor + condition), so treatment effects were estimated after adjusting for donor-specific differences. Genes with an FDR, using Benjamini–Hochberg, < 0.05 and |log2 foldchange| >1 were considered to be differentially expressed. Overrepresentation analysis was performed using clusterProfiler (36) with the full transcriptome as the background gene set.

### ROS production

NADPH oxidase activity was measured by assaying the hydrogen peroxide production from cultured neutrophils, in response to various stimuli with the Amplex Red kit (Molecular Probes, Life Technologies), as described previously (28). In short, cultured neutrophils (0.25 × 10^6^/ml) were stimulated in HEPES+ medium with opsonized *E. coli* (0.25 × 10^9^/ml), zymosan (1 mg/ml, Sigma-Aldrich), serum-treated zymosan (STZ, 1 mg/ml), PMA (100 ng/ml, Sigma-Aldrich), or PAF/fMLP (both 1 μM) in the presence of Amplex Red (0.25 μM) and horseradish peroxidase (0.5 U/ml). Fluorescence was measured at 30-s intervals for 30 min with a plate reader (infinitiPRO2000 series, Tecan). The maximum slope of hydrogen peroxide release was assessed over a 2-min interval to determine the activity of NADPH oxidase.

### Adhesion assay

A static adhesion assay was performed as previously described (28). Neutrophils were stained with Calcein AM for 30 min, washed, and subsequently resuspended in HEPES+ medium either unstimulated or stimulated with G-CSF (20 ng/ml, Neupogen, clinical grade), DTT (10 mM, Sigma-Aldrich), fMLP (1 µM, Sigma-Aldrich), C5a (10 nM, Sino Biological Inc.), or PMA (100 ng/ml) for 30 min at 37°C, 5% CO_2_ in a flat bottom 96-well MaxiSorp plate (polystyrene). Afterward, wells were washed twice with PBS, and cells were lysed with 0.1% Triton X-100. Fluorescence was measured using a plate reader (Tecan infinitiPRO2000 series).

### Chemotaxis assay

Chemotaxis assay was performed as previously described (28). Neutrophils were stained with Calcein AM for 30 min, washed, and transferred into a 3-µm FluoroBlok insert (Corning). Inserts were placed in 37°C pre-warmed 24-well plates with HEPES+ only, C5a (20 nM, Sino Biological Inc.), or fMLP (100 nM, Sigma-Aldrich). Fluorescence was measured using a microplate reader (Tecan, infinitiPRO2000 series). Measurements were performed every 45 s over 45 min. Maximum migration speed was defined as the maximum slope determined over a 2-min interval.

### Statistics

Normality was evaluated using the Shapiro–Wilk test. For data following a normal distribution, group differences were assessed using a one-way ANOVA with Tukey’s HSD test for pairwise comparison. For non-normal data, differences were tested using the Kruskal–Wallis test followed by a two-sided Dunn’s post hoc test with Benjamini–Hochberg FDR multiple testing correction. Adjusted P values were considered statistically significant below 0.05. Data are shown as mean ± SD in scatterplots and as median with interquartile range for boxplots. Sample size and the statistical tests used are reported in the corresponding figure legend. All statistical analyses were performed in R (version 4.6.1).

### Online supplemental material

Supplementary material contains additional information for sequencing, immunoblotting, flow cytometry, and RNA-seq analysis. Fig. S1 shows characterization of *FERMT3* KO human CD16^+^ cultured neutrophils. Fig. S2 shows gating strategies used for the characterization of bulk fractions of FERMT3, Cas9-only, and untreated cultured neutrophils. Fig. S3 shows effect of depleting kindlin-3 on the transcriptomic profile of cultured neutrophils. Table S1 shows gRNAs used for CRISPR-Cas9 in HSPCs producing *FERMT3* KO cultured neutrophils. Table S2 shows list of antibodies used in FACS analysis. Table S3 shows differential expression analysis of FERMT3 KO compared with untreated cultured neutrophils. Table S4 shows list of off-targets identified for each gRNA using CRISPOR, including exonic, intronic, and intergenic hits.

## Supplementary Material

Table S1 shows guide RNAs used for CRISPR-Cas9 in HSPCs producing *FERMT3* KO cultured neutrophils.

Table S2 shows list of antibodies used in FACS analysis.

Table S3 shows differential expression analysis of FERMT3 KO compared with untreated cultured neutrophils.

Table S4 shows list of off-targets identified for each guide RNA using CRISPOR, including exonic, intronic, and intergenic hits.

SourceData F1 is the source file for Fig. 1.

## Data Availability

Raw RNA sequencing data have been deposited in the Gene Expression Omnibus database (GEO) under the accession code GSE328304 for public access.
