# Supplementary material for: CRISPR/Cas9-mediated loss of FERMT3 reveals kindlin-3–independent neutrophil activity in LAD-III
Source: J Hum Immun. 2026 Oct 21;2(6):e20260086. doi: 10.70962/jhi.20260086 (PMC13685712; doi:10.70962/jhi.20260086)

SourceData Figure1B

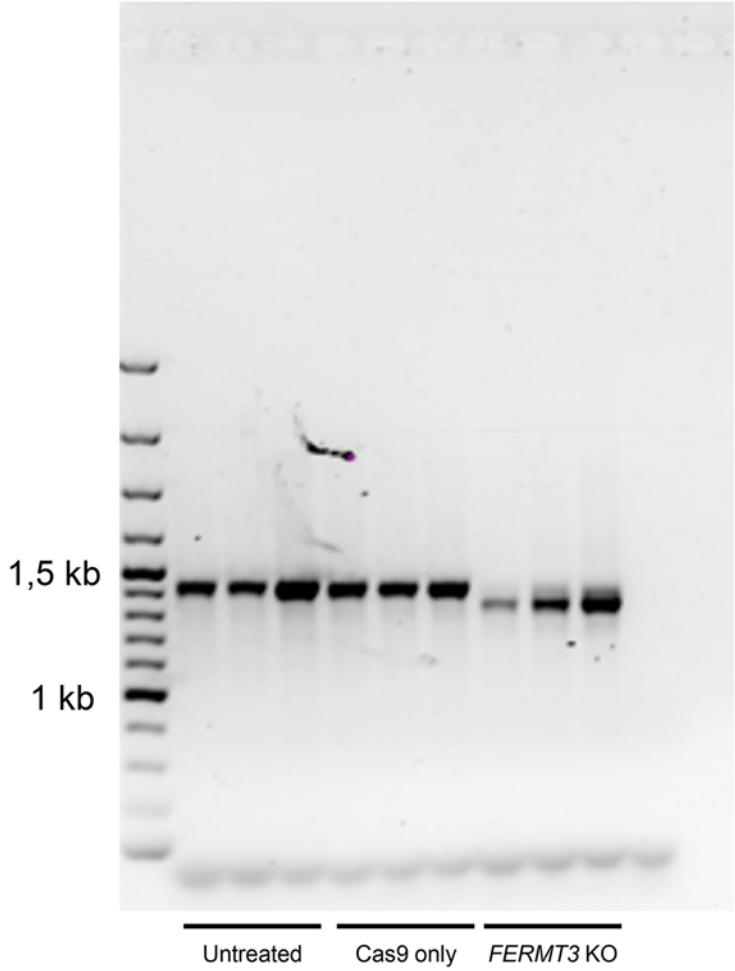

SourceData Figure1D

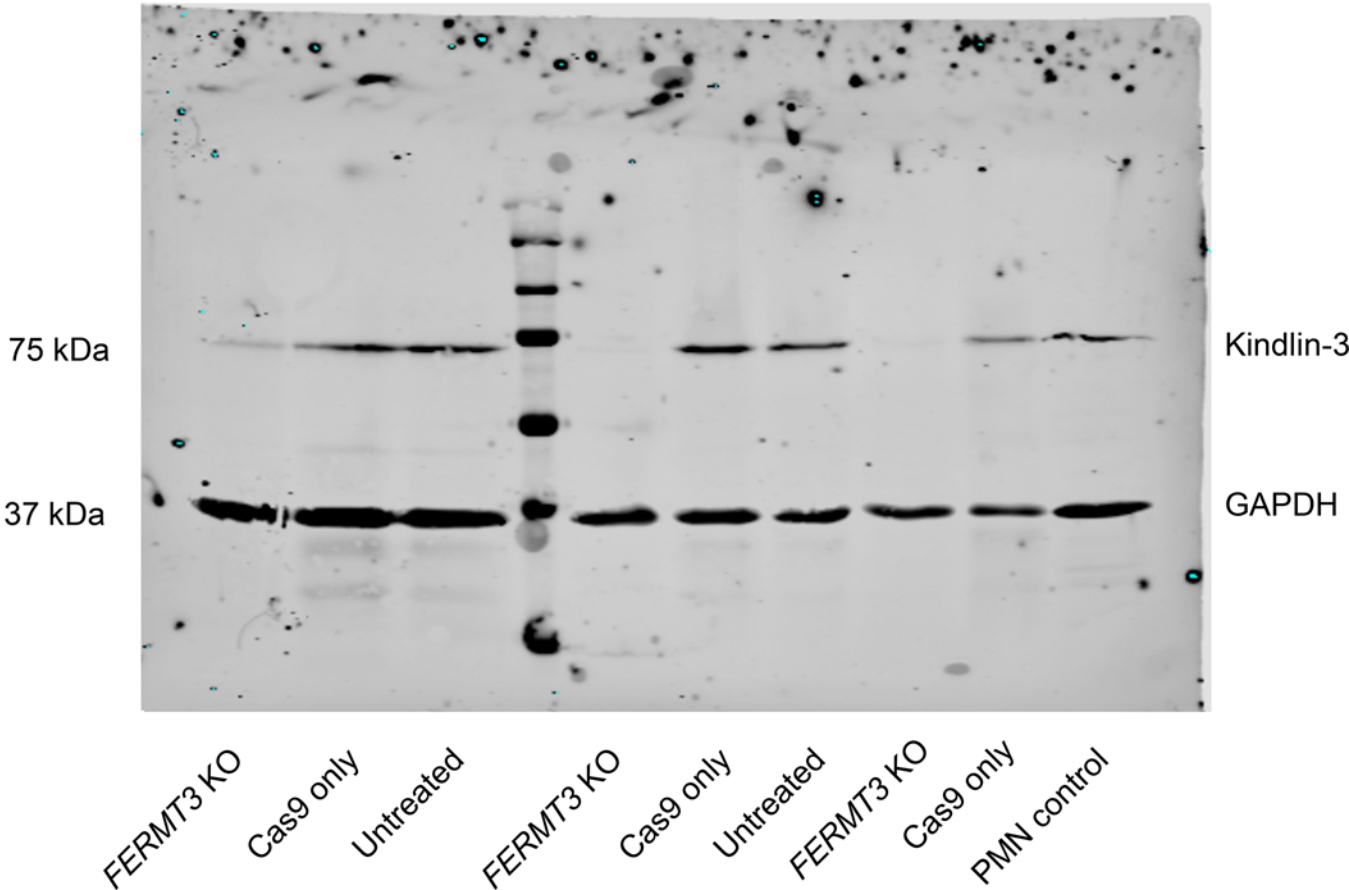

Supplement: Supplementary file 1 [file jhi_20260086_sourcedataf1.pdf]
